# The *Drosophila* Secretome

**DOI:** 10.64898/2026.08.24.746765

**Published:** 2026-10-21

**Authors:** Yanhui Hu, Nahid Ausrafuggaman, Jenna Kalina, Austin Veal, Mujeeb Qadiri, Ah-Ram Kim, Norbert Perrimon

## Abstract

Secreted proteins are synthesized within the cell and actively transported outside, where they contribute to development, physiology, immunity, and cell–cell communication. In *Drosophila melanogaster* , many secreted proteins are evolutionarily conserved, making this species a powerful model for studying human development, cancer, neurobiology, and immune regulation. To assemble the *Drosophila* secretome, we systematically analyzed all fly protein sequences with computational tools, integrating FlyBase, UniProt and Gene Ontology annotations, and incorporating large-scale proteomics datasets. We identified 5,280 genes encoding putative secreted proteins, assigned confidence ranks and scores based on the type and strength of supporting evidence, and generated an online resource ( www.flyrnai.org/apps/fly_secretome/ ) for exploring these data. Comparison with the human secretome shows that 56% of *Drosophila* secretome genes are conserved in the human genome and of these, 81% are annotated as secreted in human. In addition, comparison with *Drosophila* single-cell transcriptomic data revealed that secreted proteins are more tissue-specific than other genes. Finally, we demonstrate the utility of this resource by analyzing changes in expression of genes encoding putative secreted proteins during aging based on snRNA-seq datasets from the Aging Fly Cell Atlas.

